# Novel 3D in situ visualization of seal heartworm (*Acanthocheilonema spirocauda*) larvae in the seal louse (*Echinophthirius horridus)* by X-ray microCT

**DOI:** 10.1038/s41598-022-18418-y

**Published:** 2022-08-18

**Authors:** David Ebmer, Stephan Handschuh, Thomas Schwaha, Ana Rubio-García, Ulrich Gärtner, Martin Glösmann, Anja Taubert, Carlos Hermosilla

**Affiliations:** 1 Institute of Parasitology, Biomedical Research Center Seltersberg, Justus Liebig University Giessen, Schubertstr. 81, 35392 Giessen, Germany; 2 VetCore Facility for Research, Imaging Unit, University of Veterinary Medicine Vienna, Veterinaerplatz 1, 1210 Vienna, Austria; 3 Department of Evolutionary Biology, University of Vienna, Djerassiplatz 1, 1030 Vienna, Austria; 4 Sealcentre Pieterburen, Hoofdstraat 94a, 9968 AG Pieterburen, The Netherlands; 5 Institute of Anatomy and Cell Biology, Justus Liebig University Giessen, Aulweg 123, 35385 Giessen, Germany

**Keywords:** Entomology, Parasitic infection, 3-D reconstruction

## Abstract

The seal heartworm *Acanthocheilonema spirocauda* (Nematoda: Onchocercidae) parasitizes the heart and pulmonary arteries of various phocid seals of the Northern Hemisphere. Over many decades, potential vectors of this parasite have been discussed, and to this date, the life cycle is not fully known. The seal louse *Echinophthirius horridus* (Anoplura: Echinophthiriidae) is an obligatory, permanent and haematophagous ectoparasite of phocids that has been hypothesized to function as obligate intermediate host for *A. spirocauda*. We examined 11 adult *E. horridus* specimens collected from stranded harbour seals (*Phoca vitulina*) in rehabilitation at the Sealcentre Pieterburen by X-ray microCT imaging, aiming to illustrate larval *A. spirocauda* infection sites in situ. In three of these specimens, thread-like larvae were detected in insect organs. Detailed imaging of the most infected louse revealed a total of 54 *A. spirocauda* larvae located either in fat bodies or the haemocoel. Histological analysis of the same specimen illustrated nematode cross-sections, confirming X-ray microCT data. The current data strongly suggest that *E. horridus* is a natural intermediate host for *A. spirocauda*. Moreover, we demonstrate the potential of X-ray microCT-based imaging as a non-destructive method to analyze host-parasite interactions, especially in the neglected field of marine mammal parasitology.

## Introduction

*Acanthocheilonema spirocauda* (Leidy, 1858) Anderson, 1992 is an angiotropic marine parasitic nematode of the family Onchocercidae (superfamily Filarioidea)^1^ that parasitizes a broad range of phocid seal species in the Northern Hemisphere, including harbour seals (*Phoca vitulina*), ringed seals (*Pusa hispida*) and the endangered Mediterranean monk seals (*Monachus monachus*)^2,3^. In the final hosts, adult *A. spirocauda* mainly reside in the right ventricle and atrium of the heart and in the pulmonary arteries, reaching a body length of more than 20 cm^1–6^. Epidemiological studies on *A. spirocauda* infections in free-ranging *P. vitulina* in the Wadden sea showed varying prevalences (32%^7^, 25%^4^, 6%^5^ and 4%^6^). *A. spirocauda* infections are predominantly reported in immature seals^4–6,8,9^ and do not seem to significantly affect the health status of the animals^5,6^. However, low prevalences of *A. spirocauda* infections in older seals^4,5^ could result from a high mortality in severely infected young seals^1,10^. *A. spirocauda*-associated lesions have been described in parasitized hearts, even with fatal consequences^5,10^. For example, Lehnert et al.^5^ reported a perforation of the right atrium with marked pericardial effusion as the cause of death in a severely infected harbour seal.

**Figure 1:**
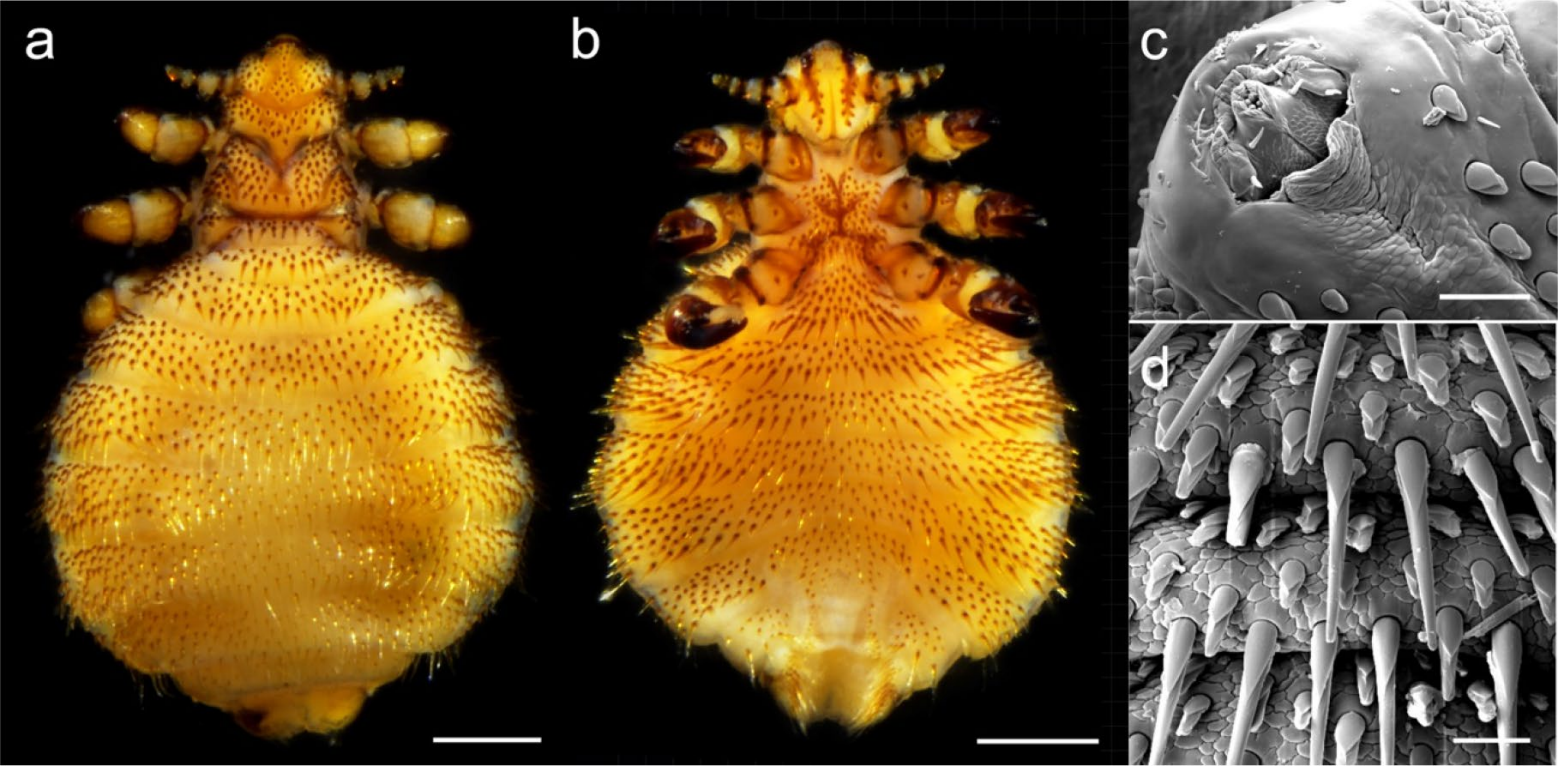
Stereomicroscopic and scanning electron microscopic (SEM) images of the seal louse, *Echinophthirius horridus*. (**a**) Dorsal and (**b**) ventral view of the heavily infected female louse with a plump gravid abdominal area. (**c**) Eversible proboscis evolved for haematophagous life style. (**d**) Modified setae as sensorial organs located on the cuticular. Scale bars: (**a**, **b**) 500 µm, (**c**, **d**) 50 µm.

Over many decades, transmission pathways and potential natural intermediate hosts of *A. spirocauda* have been discussed, however, the full life cycle is not yet completely known (for a review, see Leidenberger et al.^1^). Adult female *A. spirocauda* are viviparous and release microfilariae into the bloodstream of final hosts^1^. Diaplacental transmission of microfilariae has been suggested, but due to low infection rates in adult seals this pathway does not seem to play a major role^1^. Consistently, it was hypothesized that the haematophagous marine seal louse, *Echinophthirius horridus* (von Olfers, 1816) Fahrenholz, 1919 (Anoplura: Echinophthiriidae) (Fig. 1), might be the natural obligate intermediate host of this filarioid nematode^1,11^. Representatives of the family Echinophthiriidae are obligatory and permanent ectoparasitic insects^12^ and, in contrast to terrestrial anoplurans, they are highly adapted to marine environments^13^. While various echinophthiriid species share a strict host specificity, the seal louse *E. horridus* shows a rather broad host range by infesting eight different phocid seal species, including harbour seals and ringed seals^12^. Similar to *A. spirocauda*, *E. horridus* is also restricted to the Northern Hemisphere^12^ and mainly occurs in immature seals^6^. Due to their obligate and permanent haematophagous feeding habits, echinophthiriid species are likely to transmit and spread vector-borne bacteria, such as *Anaplasma phagocytophilum*, *Bartonella henselae*, *Mycoplasma *sp., *Rickettsia* sp., and *Salmonella enteritidis* in free-living pinniped populations^14–17^.

Filarioid nematodes affecting humans and terrestrial mammals all share obligate heteroxenous life cycles involving haematophagous ectoparasites as intermediate hosts^18^. Within the genus *Acanthocheilonema,* most representatives parasitize subcutaneous tissues and abdominal cavities of mammalian hosts, and a broad range of competent arthropod vectors has been reported, including ticks (e.g. *Ornithodorus tartakovskyi* for *A. vitae* in rodents^19^), louse flies (e.g. *Hippobosca longipennis* for *A. dracunculoides* in dogs^20^) and even chewing and anopluran lice (e.g. *Heterodoxus spiniger* and *Linognathus setosus* for *A. reconditum* in dogs^21^). Nonetheless, very little is known on intermediate hosts of filarioid parasites circulating in marine mammals^1,17,22^.

Since early studies from the nineteen sixties/seventies failed to detect *A. spirocauda* stages in *E. horridus*, *Culex pipiens* mosquitoes and simuliid blackflies were consequently assumed as potential vectors^10,23^. In 1981, Geraci et al.^24^, for the first time, succeeded to detect different larval stages of *A. spirocauda* in dissected *E. horridus* specimens. They found *A. spirocauda* larvae in 70 of 102 lice obtained from one stranded *P. vitulina*, which also showed microfilaremia. The authors described four larval stages [microfilariae, first-stage larvae (L1), second-stage larvae (L2) and third-stage larvae (L3)], which differed in size and morphology and were localized in the fat body, the gastrointestinal tract, the haemocoel, the claws and the head of dissected lice specimens^24^. In the following years, Dailey and Fallace^25^ presented a significant correlation between natural seal heartworm infections and seal lice infestations in harbour seals and Lehnert et al.^6^ found a single *A. spirocauda* larva during dissection of 35 adult seal lice. The recent molecular diagnosis of *A. spirocauda* in *E. horridus* lice strongly supported the hypothesis of *E. horridus* vector capacity^17,26^. Nevertheless, the study of Geraci et al.^24^ constitutes the most detailed analysis on different larval stages and their development in *E. horridus* lice, thereby representing the most convincing support of this hypothesis until today.

For the detection of protozoan and metazoan parasites inside arthropod tissue, ectoparasites can be screened in different ways, including dissections^6,20,21,24^, pathohistological examinations^27^, and the use of molecular methods^17,26^. In recent years, X-ray microCT imaging technique as a non-invasive and non-destructive method has been applied to reveal new three-dimensional insights into host-parasite relations^28–31^. However, up to date, studies in medical and veterinary parasitology using this imaging technique are rare^32^. Therefore, in this study, we aimed to contribute to explore the role of *E. horridus* as an intermediate host of *A. spirocauda* by using 3D X-ray microCT imaging. Using this non-destructive technology, we screened adult *E. horridus* specimens from naturally infested harbour seals during their rehabilitation at Sealcentre Pieterburen for the presence and in situ localization of different larval stages of *A. spirocauda*. The three-dimensional visualization of different larval stages and their tissue-related assignment should help to clarify distinct steps within the heteroxenous life cycle of *A. spirocauda* and the general role of *E. horridus* as intermediate host within marine ecosystems.

## Results

For X-ray microCT imaging, a total of 11 adult *Echinophthirius horridus* lice were selected out of a pool of various preserved specimens (*n* = 53), which all originated from different, originally free-living, stranded harbour seals during their rehabilitation period at the Sealcentre Pieterburen, the Netherlands. Thus, the actual infection status of preserved *E. horridus* specimens was unknown. Moreover, no information on the *Acanthocheilonema spirocauda* infection status of the seals (or on the presence of microfilaremia) was available at the time of experimental setting initiation. However, Hirzmann et al.^17^ meanwhile proved the presence of *A. spirocauda*-DNA in the same pool of sampled *E. horridus* lice. The individual selection of adult female specimens for 3D imaging was based on morphological aspects (plump abdominal area, typical two pairs of gonopods, and a body length of more than 2.5 mm) to increase the chance of screening lice, which had extensively sucked seal blood.

### X-ray microCT analyses and 3D reconstruction

In a first experiment, 11 female *E. horridus* were screened to obtain an overview on arthropod morphology as well as the anatomical features, location and measures of organs. Since this study constitutes, to our best knowledge, the first X-ray microCT imaging analysis of echinophthiriid lice, measurements and morphological details of various organs of different *E. horridus* specimens were compared. Taking into account known microhabitats of *A. spirocauda* larvae inside *E. horridus*^24^, particularly subcuticular fat bodies and the haemocoel of lice were carefully inspected. We found irregularities in fat body densities in three of 11 *E. horridus* specimen (27%). By 3D reconstruction, these heterogeneous inclusions in fat bodies were reconstructed as thread-like, often sausage-shaped structures clearly discriminable from surrounding insect tissue. Based on the molecular genetic evidence of *A. spirocauda* in the same pool of sampled seal lice^17^ as main diagnostic and identification criterion, these structures were interpreted as *A. spirocauda* larvae.

**Figure 2:**
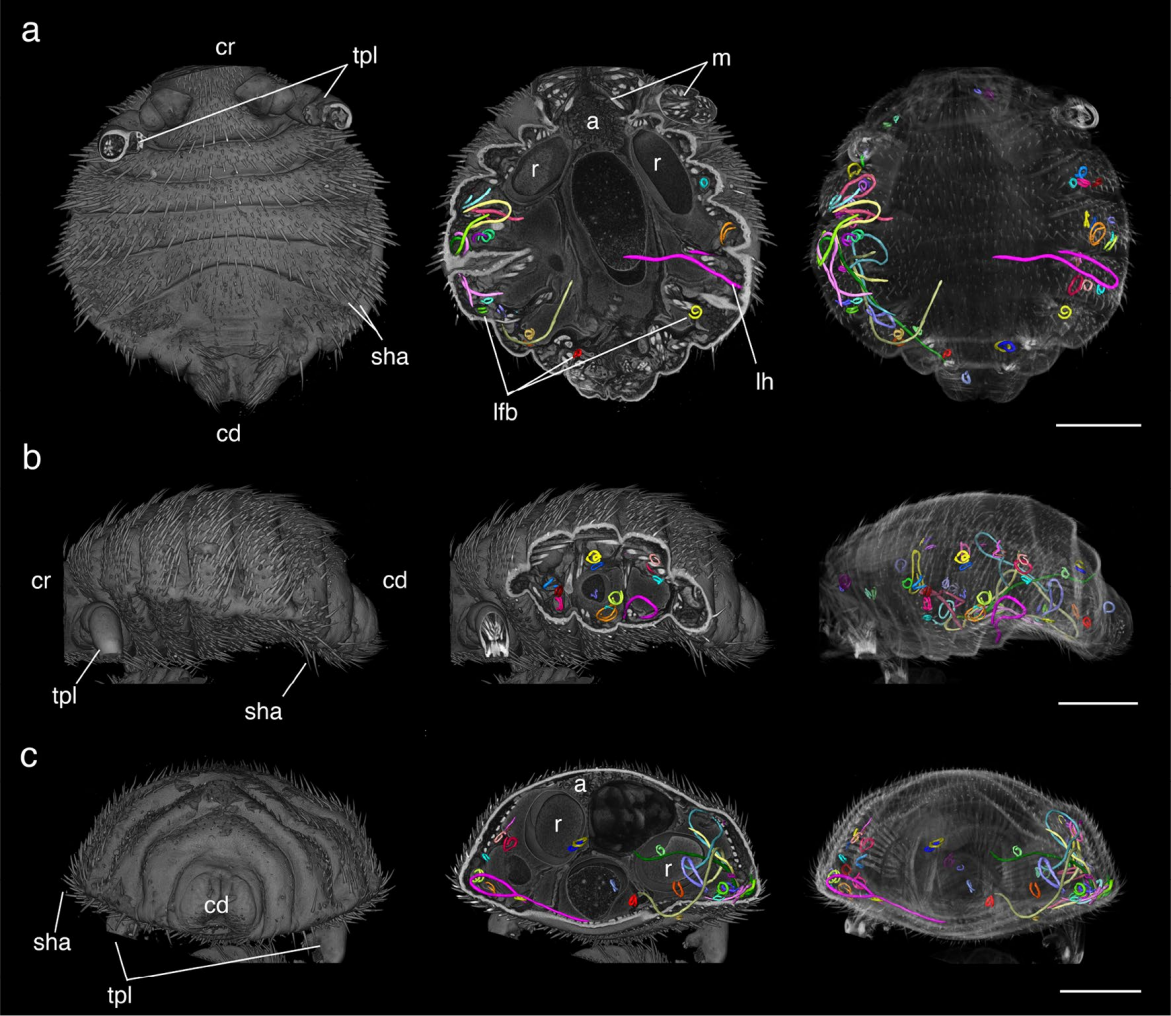
X-ray microCT imaging analysis and 3D reconstruction of *Echinophthirius horridus*, with various *Acanthocheilonema spirocauda* larvae (colored, string to sausage-shaped structures) in different body layers. In situ visualization. (**a**) Ventral view. (**b**) Lateral view (**c**). Caudal view. Scale bars: 500 µm. Abbreviations: a = alimentary tract, cd = caudal, cr = cranial, lfb = larvae contorted in the fat body, lh = larvae located in the haemocoel, m = musculature, r = reproductive tract (nits/eggs), sha = setae modified to hairs, tpl = third pair of legs.

**Figure 3:**
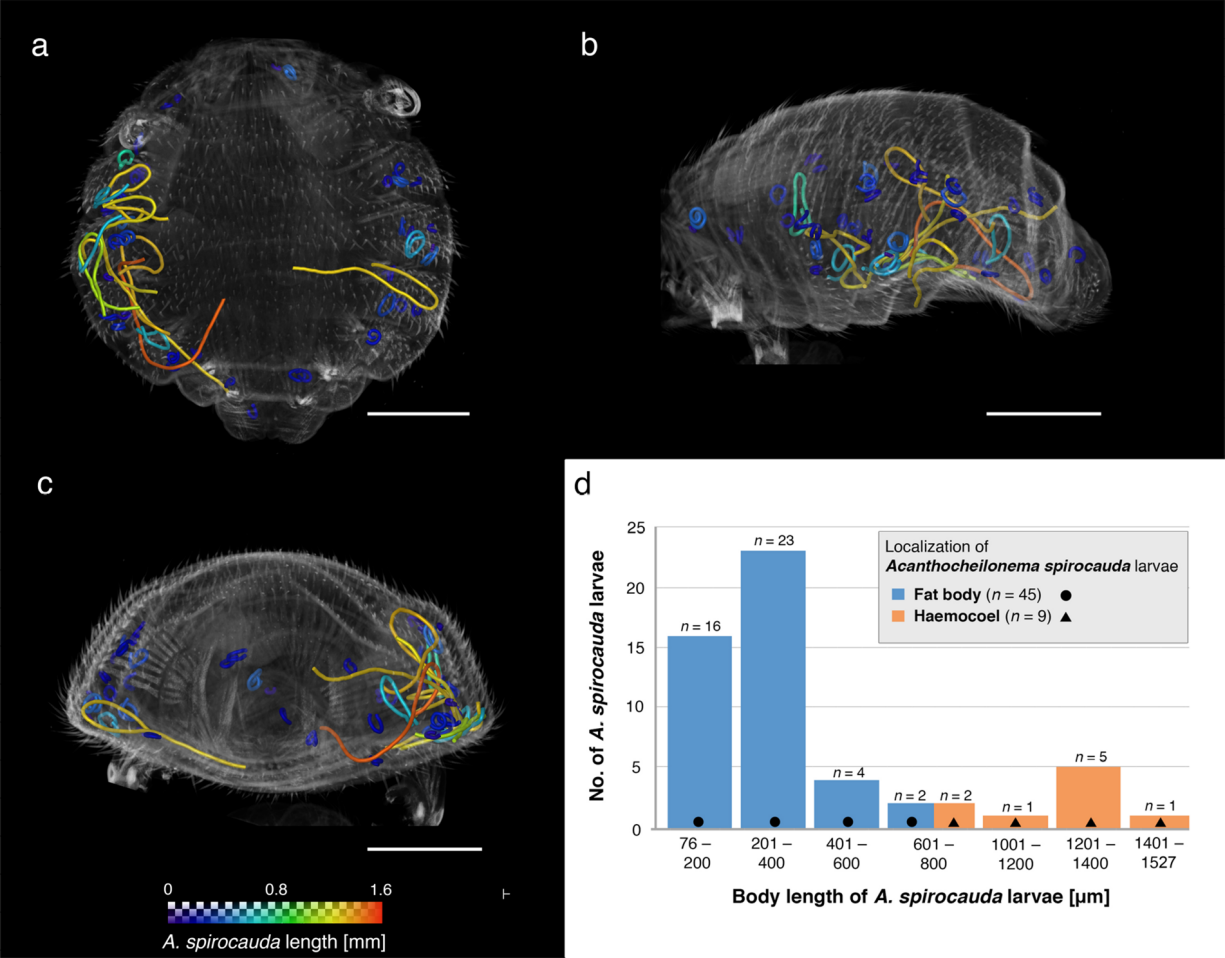
3D reconstruction of *Echinophthirius horridus* based on X-ray microCT showing *Acanthocheilonema spirocauda* larvae ordered by body length. (**a**) Ventral view. (**b**) Lateral view. (**c**) Caudal view. (**d**) Overview of larval localization in relation to body length. Scale bars: 500 µm.

**Figure 4:**
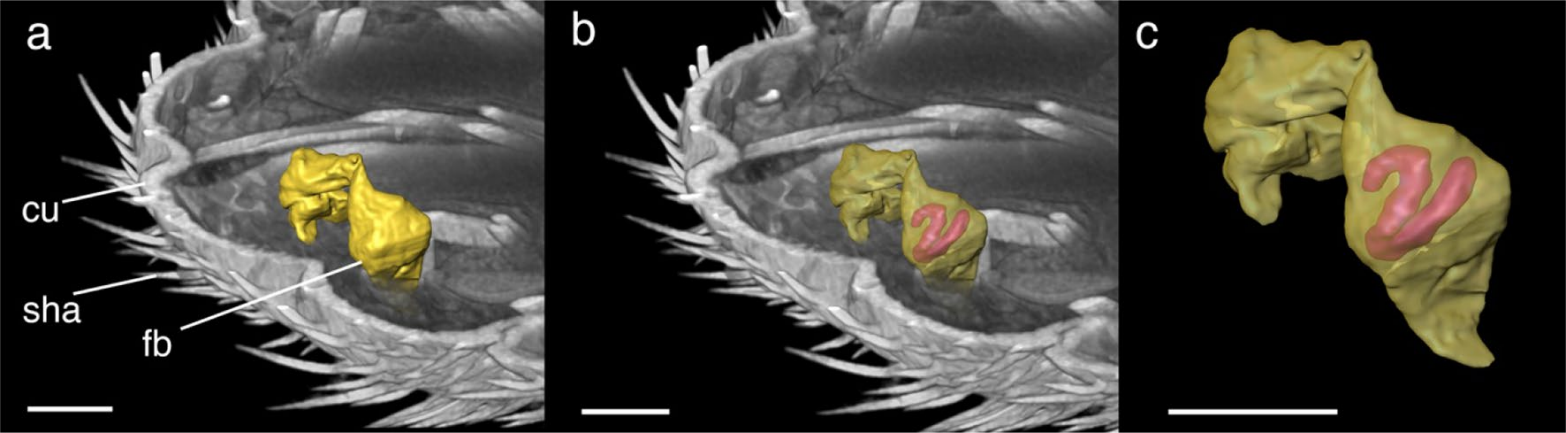
3D reconstruction of fat body element of *Echinophthirius horridus*. (**a**) Yellow marked fat body, (**b**) revealing a plump, sausage-shaped *Acanthocheilonema spirocauda* larva (potentially L1-stage) inside. (**c**) Isolated fat body, containing *A. spirocauda* larva. Scale bars: 100 µm. Abbreviations: cu = cuticle, fb = fat body, sha = setae modified to hairs.

In the second phase of this study, one female *E. horridus* individual presenting conspicuous larval structures in insect organs was subjected to high resolution imaging (Figs. 2, 3; Supplementary Video 1). In total, 54 *A. spirocauda* larvae ranging from 76 to 1527 µm total body length were detected, of which 45 larvae (83%) were found enclosed and encapsulated in fat bodies and measured between 76 and 779 µm in length. Only two fat-body localized larvae reached a body length over 600 µm (Fig. 3). In general, *A. spirocauda* larvae present in fat bodies appeared strongly contorted and showed a typical sausage-shape (Fig. 4). Nine larvae (17%) were located freely in the haemocoel and showed body lengths of 615–1527 µm (Fig. 3). Overall, the longest larval stages showed a body length of > 1000 µm (1122–1527 µm, *n* = 7) and were exclusively located in the haemocoel of the seal louse.

### Histology

**Figure 5:**
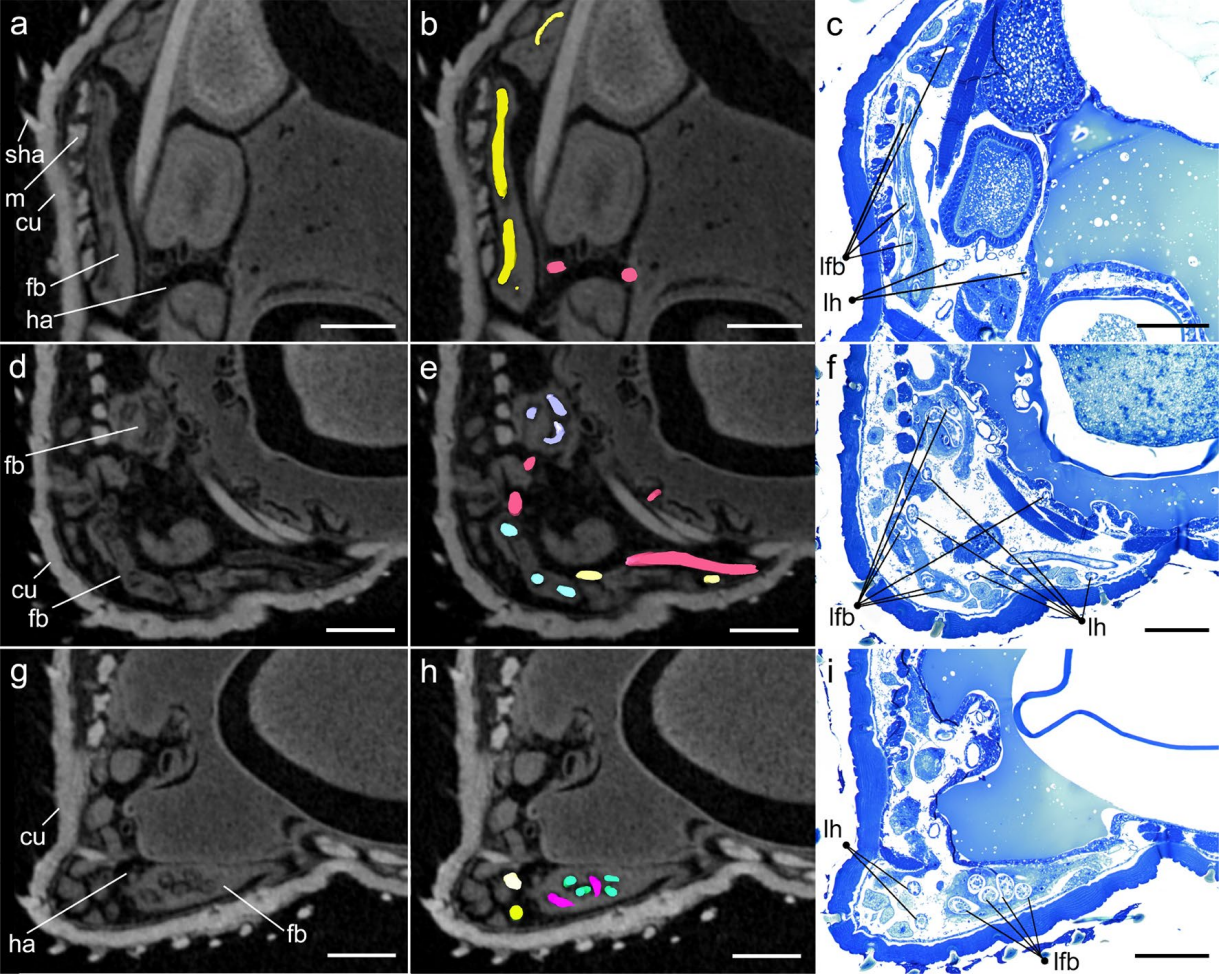
X-ray microCT images with marked larval structures and histological cross-sections. Scale bars: 100 µm. Abbreviations: cu = louse cuticle, fb = fat body, ha = haemocoel, lfb = larvae contorted in the fat body, lh = larvae located in the haemocoel, m = musculature, sha = setae modified to hairs.

**Figure 6:**
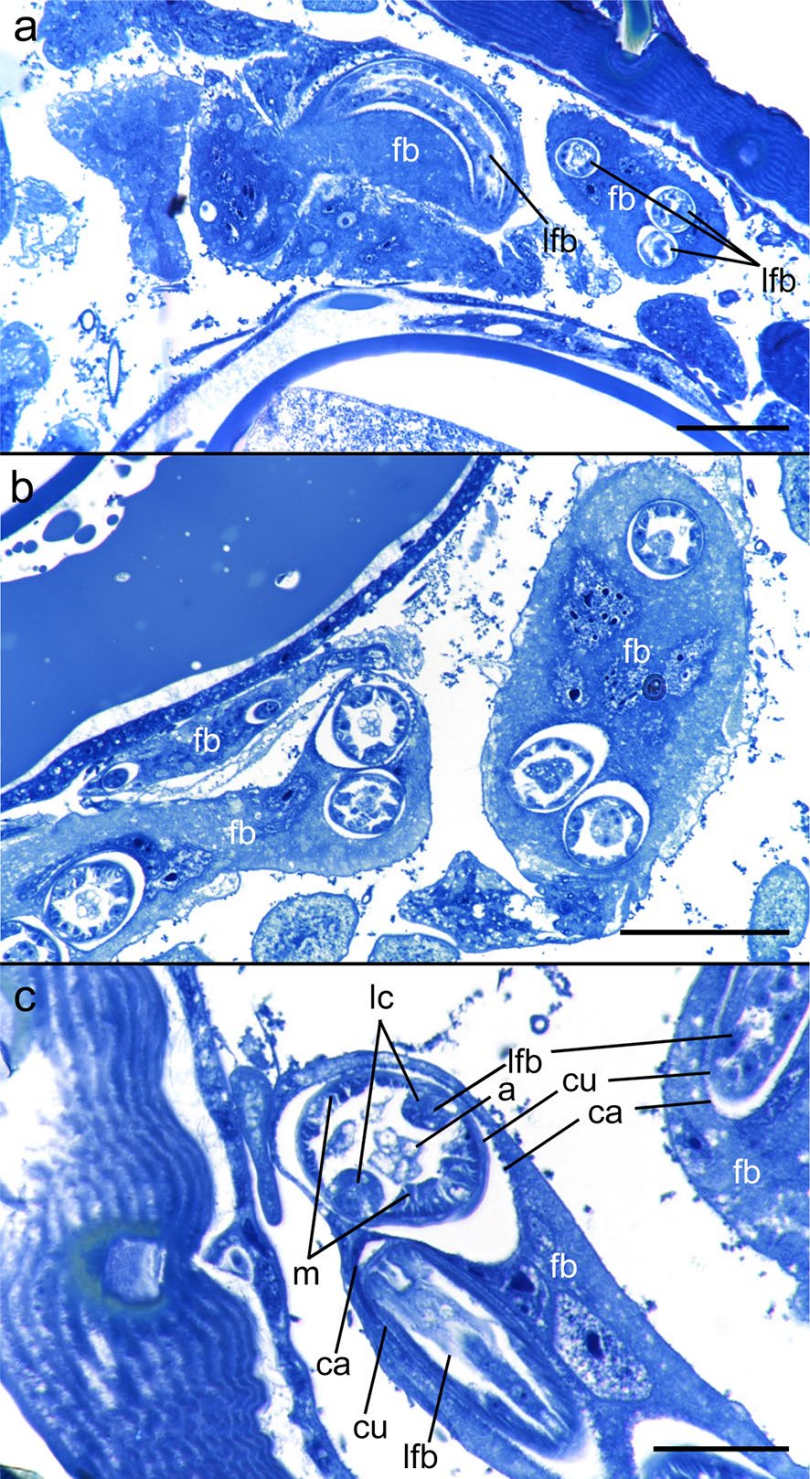
Histological cross-sections showing multiple *Acanthocheilonema spirocauda* larvae encapsulated in fat bodies. Scale bars: (**a**, **b**) 50 µm, (**c**) 20 µm. Abbreviations: a = nematode alimentary tract, ca = capsule, cu = nematode cuticle, fb = fat body, lc = nematode lateral chords, lfb = larvae contorted in the fat body, m = nematode musculature.

To confirm that thread-like structures observed by X-ray microCT imaging in fact were *A. spirocauda* larvae, the heavily infected *E. horridus* individual was subjected to histological analysis. Using semi-thin sections, conspicuous areas in *E. horridus* fat bodies and haemocoel indeed revealed multiple capsule-like structures containing nematode cross-sections. Nematode cuticula and inner larval organs could be identified, including alimentary tract, musculature and lateral chords (Figs. 5, 6), thereby delivering final evidence on the presence of *A. spirocauda* larvae inside *E. horridus* and confirming X-ray microCT analyses.

## Discussion

To date, only few studies suggested a role of *Echinophthirius horridus* as obligate intermediate host in the heteroxenous life cycle of *Acanthocheilonema spirocauda.* This was based on the morphological detection of different larval nematode stages (i. e microfilariae, L1, L2, L3) during dissections of *E. horridus* specimens^6,24^, a significant statistical correlation between infestations with seal lice and infections with heartworms in harbour seals^25^, and a molecular xeno-monitoring of *A. spirocauda* in *E. horridus*^17,26^. For the first time, we here used X-ray microCT imaging to three-dimensionally visualize stages of *A. spirocauda* larvae in their in situ microhabitats inside the seal louse. Thereby, larval stages exhibiting different length and localizations were detected, showing that this nematode parasite is capable to develop into up to 1527 µm long stages inside the seal louse. Our results support the role of *E. horridus* as an obligate intermediate host of *A. spirocauda* in the marine ecosystem, thereby playing an essential role in pathogen transmission and maintenance of acanthocheilonemosis in free-ranging seal populations.

Using non-invasive X-ray microCT imaging, various worm-like structures inside different organs of *E. horridus* were illustrated. Histological examination of the same specimen confirmed X-ray microCT-based diagnosis and proved that these thread-like structures in the fat body and haemocoel were indeed nematode larvae. Thereby, the identification of detected nematodes as *A. spirocauda* was conducted on the basis of following knowledge: (i) *E. horridus* specimens analyzed in this study were sampled during a monitoring study on vector-borne pathogens in seal lice and the presence of *A. spirocauda*-DNA in the same pool of *E. horridus* specimens has been confirmed^17^. Beside the development of a specific nested PCR for the detection of *A. spirocauda*, Hirzmann et al.^17^ screened various *E. horridus* pools using pan-filarial primers for a broad spectrum of filarial nematodes^33^ and no other filarioid species or mix-infections could be detected^17^ Thereby, this molecular genetic evidence was used as main diagnostic criterion for identification of *A. spirocauda* in the present study. (ii) *A. spirocauda* is a common endoparasite of harbour seals (*Phoca vitulina*) and the only described angiotropic filarioid nematode species infecting harbour seals in the Wadden sea^4–7^. (iii) *Dirofilaria immitis*, another filarioid nematode^18^, was described in *P. vitulina* before^34^, but has never been detected in this host in the Wadden sea. Additionally, *D. immitis* is well known to be transmitted by mosquitoes^35^ and has never been detected in echinophthiriid lice^17^.

The potential of X-ray microCT as a method to visualize and analyze host-parasite interactions in a non-invasive and non-destructive manner has already been demonstrated by other studies^28–31^. We highlight these advantages on the example of an echinophthiriid louse, to our best knowledge, for first time in this study. Thereby, our data clearly show that non-invasive 3D imaging reveals insights into detailed anatomical structures of an insect ectoparasite and its potential pathogen fauna, while completely preserving insect morphology. However, distinct analyzation of (inner) larval morphology, stage classification or even species identification is not possible with this method. Utilizing the non-invasive and non-destructive aspect of this imaging technology, these disadvantages and limitations could be equalized by combining X-ray microCT with other diagnostic methods, like dissections of lice and exact morphological identification and characterization of larvae via light microscopy. So far, X-ray microCT analyses have rarely been used in medical and veterinary parasitology^32^. Moreover, the vector role of most echinophthiriid species constitutes a neglected research topic in the field of marine mammal parasitology worldwide^17^, even though their obligatory, permanent, and haematophagous *modus vivendi* is eminently suitable for pathogen transmission. We demonstrate the potential of non-invasive 3D X-ray microCT imaging for pathogen screening in ectoparasites, which may be applied to a broad spectrum of investigations in veterinary and medical entomology.

Geraci et al.^24^ provided the strongest evidence for *E. horridus* as an intermediate host for *A. spirocauda*. They detected *A. spirocauda* larvae in 70 of 102 dissected seal lice and classified these larval stages as microfilariae, L1, L2 and L3, based on body length and other morphological features^24^. Overlapping lengths in reference measurements and highly variable larval lengths in the current study did not allow for developmental stage identification in this analysis. Furthermore, distinct morphological parameters used for larval stage identification like a cephalic knob in L1, distinct inner structures like oesophagus in L2, or papilla-like structures at the posterior end of L3^24^, could not be detected by the employed imaging technique, thus hampering stage identification. Interestingly, in the current study, 16 larvae (ranging from 76–195 µm in length) were shorter than 197 µm, a measured value defined as minimal body length of *A. spirocauda*-L1 by Geraci et al.^24^. L1-stages were also defined as “sausage” stage^24^, a term which is known from other filarioid nematodes as well, e.g. *D. immitis*^35^. However, the larval length may also have been influenced by fixation or staining. For example, after *E. horridus* dissection, Geraci et al.^24^ kept larval stages in physiological saline, while *E. horridus* specimens in this study were fixed in 80% ethanol and stained with Lugol’s solution. Both compounds are known to cause tissue shrinkage. Because our data were not corrected for shrinkage, the presented larval length data should be interpreted with caution. However, based on our data, X-ray microCT imaging constitutes a suitable tool to detect short larvae, which could potentially be overlooked or damaged during stereomicroscopy-guided dissections of lice. Moreover, Geraci et al.^24^ measured 2% formalin-fixed microfilarial stages obtained from a microfilaremic harbour seal^24^. The stages were exclusively found in the gastrointestinal tract of the examined specimens^24^. Based on these observations, it is possible that larval stages located in fat bodies, as found in the current study, are L1-stages.

Demonstrating a high *A. spirocauda* larval burden (54 larvae in a single female louse), the current data highlight the enormous role of echinophthiriid lice for effective pathogen transmission. Compared to data from Geraci et al.^24^, the specimen analyzed in the present study represents a highly infected seal louse. With respect to mean parasite burden, Geraci et al.^24^ reported that 87% of infected *E. horridus* harboured 4.6 L1 stages, 54% showed 3.0 L3 stages, and 26% showed 1.4 L2 stages. Further, the authors described the fat body of the seal louse as main localization of *A. spirocauda* larvae, however, larvae were also found in the gastrointestinal tract, haemocoel, claws and head of *E. horridus* specimens^24^. In the present study, we found larval structures exclusively in fat bodies and the haemocoel of *E. horridus.* Six larvae revealed a body length of > 1170 µm and, when following the classification criteria by Geraci et al.^24^, may be classified as L3. Interestingly, these large and coiled larvae were exclusively found in the haemocoel.

Including previous reports of *A. spirocauda* in seal lice^6,17,24,26^ and data of other filarioid parasites^35–38^, the results of the current study contribute to further unravel the life cycle of this nematode in its proposed intermediate host. After *A. spirocauda* ingestion by *E. horridus* via a blood meal, microfilariae necessarily have to leave the alimentary tract for further development, as it is well-known for other filarioid nematodes, e.g. *D. immitis*, *D. repens,* or lymphathic filarial species^35–37^. Otherwise, non-migrating stages will be excreted by insect host. While penetrating middle gut membranes, micro-lesions and tissue damage could occur, as it was reported for membrane lesions in the vector *Aedes aegypti* caused by microfilariae of lympathic filariae *Brugia malayi*^39^. For *Dirofilaria* spp., Malpighian tubules of mosquito vector constitute favored migration site of L1 and L2 stages^35^, while thoracic musculature was reported as preferred microhabitat for lympathic filarial parasites after migration from the middle gut^37,38^. Fat bodies of *E. horridus* seem to represent a preferred microhabitat for, especially early, *A. spirocauda* larval stages, as it was reported by Geraci et al.^24^. This effect was also shown in the current study, revealing that the majority of larvae (83%) were localized in fat bodies, while only two of them reached a body length over 600 µm. Similar to other filarioids, it has been shown that *A. spirocauda* stages perform enormous length growth in its potential vector. Geraci et al.^24^ reported on up to the tenfold length of infectious L3 stages compared to ingested microfilariae/L1 stages. Considering very short stages detected in the current study, the longest larva in the current study (1527 µm) reached the 20-fold length of the smallest stage located in the fat body (76 µm). However, for transmission to seals during *E. horridus* haematophagy*,* larvae have to further migrate into the louse proboscis as it is known for other vectors^35^. As it was reported for lympharial filarial taxa^38^, L3 stages of *A. spirocauda* were described as very active with vigorous movements^24^, indicating an effective migration mobility out of abdominal area in the louse proboscis for transmission to seal final host.

In the past, low prevalences of *E. horridus* infestations (4%^5^ and 3%^6^) and *A. spirocauda* infections (6%^5^ and 4%^6^) in stranded harbour seals in the German Wadden sea have been reported. However, Lehnert et al.^6^ reported a distinct prevalence increase of both pathogens in stranded harbour seals in the last year of the study (2013) that calls for further monitoring. Since most studies reporting on low *E. horridus* prevalences^5,6^ or even no ectoparasite findings^4,7,8^ are based on necropsies of stranded harbour seals, it should be considered, that lice could leave their host after death^40^. Thereby, future prevalence studies based on examinations of free-living or stranded seals during rehabilitation periods in wildlife rescue centers could help to better understand the in vivo-occurrence of this ectoparasitosis.

Among echinophthiriid lice, only few species have been examined for their vector function so far^14–17^. Up to date, *E. horridus* has been the only echinophthiriid species suspected to function as intermediate host for a metazoan parasite species, namely the filarioid nematode *A. spirocauda*^1,24^. Nonetheless, another echinophthiriid species may also be involved in *A. spirocauda* life cycle as well, such as *Lepidophthirus piriformis*, which specifically infests Mediterranean monk seals in Europe. This seal species was recently recorded as new host for *A. spirocauda*, since two adult nematodes were found in the right ventricle of a stranded animal^3^. However, no ectoparasites were detected in this case report^3^. Another closely related marine filarioid nematode, namely *A. odendhali,* parasitizes within intermuscular fasciae and body cavities of otariid pinnipeds, such as the California sea lion (*Zalophus californianus*), the Steller sea lion (*Eumetopias jubatus*) and the Northern fur seal (*Callorhinus ursinus*). It is mainly considered as non-pathogenic but will also include haematophagous invertebrates as intermediate hosts^22,41^. However, the life cycle of *A. odendhali* is entirely unknown, whereby blood-sucking ectoparasites have been suggested as obligate intermediate host^22,41^. Moreover, California sea lions and Steller sea lions are final hosts for the echinophthiriid louse *Antarctophthirus microchir*^12^, which, so far, has never been examined for its potential role in pathogen transmission. Interestingly, free-ranging and captive California sea lions, as well as other otariids and phocids, also revealed susceptible for infections with *D. immitis*^34,41^. Based on the broad range of transmission hypotheses, future studies on the vector role of other echinophthiriid species are urgently needed to extend the current knowledge on arthropod-borne diseases in pinniped species within the highly complex marine environment.

## Methods

### Sampling

*Echinophthirius horridus* specimens were sampled in the framework of a past large-scale monitoring study on vector-borne pathogens in the Sealcentre Pieterburen, Netherlands, in mid 2012^17^. Sampling exclusively took place during routine clinical diagnostic procedures on stranded harbour seals, which were admitted for a rehabilitation period. Using lice combs and forceps, *E. horridus* specimen were removed from the seal fur and thereafter immediately stored in 80% EtOH for further investigations. Admission and rehabilitation procedures of different seal species at the Sealcentre Pieterburen were authorized by the government of the Netherlands (permission ID at time of sample-taking: FF/75/2012/015).

### Sample preparation

Sample preparation included iodine staining and mounting of lice and followed established protocols^42^ with minor modifications. Lice were fixed in 80% EtOH. Prior to staining, specimens were washed in distilled water twice for one hour to remove ethanol remnants. Thereafter, lice were placed in 1% Lugol's I_2_KI solution (Gatt-Koller, Absam, Austria) for 24 h. Finally, specimens were washed twice in distilled water for one hour.

For mounting, 200 μl pipette tips (Greiner Bio-One, Frickenhausen, Germany) were heat-sealed at the tip and filled with low-melting agarose (Carl Roth, Karlsruhe, Germany). Agarose was heated until viscosity allowed filling the pipette tip. Subsequently, stained lice were mounted into the pipette tip (3–4 specimen per tip) using dissection needles. Tight distances between specimens were important in order to image four specimens within one field of view, while points of contact were avoided.

### X-ray microCT imaging and analysis of microCT datasets

All microCT scans were acquired using an XRadia MicroXCT-400 (Carl Zeiss X-ray Microscopy, Pleasanton, CA, USA). In a first step, overview scans were acquired using the 4X detector assembly (no detector binning) with an X-ray source voltage of 60kVp and a current of 133 µA. The emitted X-ray spectrum was filtered with the Zeiss LE2 filter. Projection images were recorded over a 360° rotation with an exposure time of 30 s and an angular increment of 0.225° between projections. The FOV of overview scans was roughly 5 × 5 mm, and covered all louse specimens in the pipette tip. Isotropic voxel resolution in reconstructed volumes of overview scans varied between 2.24 µm and 2.69 µm. Based on the analysis of overview scans, a high-resolution scan from the abdomen of one specimen was acquired using the 10X detector assembly (no detector binning) with an X-ray source voltage of 40kVp and a current of 200µA (no X-ray filter). Projection images were recorded over a 360° rotation with an exposure time of 45 s and an angular increment of 0.225° between projections. Isotropic voxel resolution in the reconstructed volume of the high-resolution scan was 1.09 µm. All image volumes were saved in DICOM format.

Overview scans were inspected for the presence of nematode larvae using FIJI imageJ^43^. The high-resolution scan of one louse abdomen was imported into the 3D software package Amira 2019.4 (FEI SAS, part of Thermo Fisher Scientific). For noise reduction, the image volume was filtered with two steps of 3D bilateral filtering (step 1: kernel = 3 × 3 × 3, similarity = 20,000; step 2: kernel = 3 × 3 × 3, similarity = 40,000) followed by one step of 3D Gaussian filtering (kernel size factor = 2, standard deviation = 1). Nematode larvae were manually segmented using the *Segmentation Editor*. Based on the segmentation masks, the *Distance-Ordered Thinner* tool (Len of Ends = 8) followed by the *Trace Lines* tool were used for skeletonization in order to extract a spatial graph for each larva. Spatial graphs were smoothed using the *Smooth Line Set* tool (smoothing = 0.8, adhere to original data = 0.05, number of iterations = 100). Finally, we used the *Spatial Graph Statistics* tool to measure lengths of spatial graphs. For visualization, we used both surfaces generated from the segmentation masks together with volume rendering of the louse abdomen, as well as a visualization of spatial graphs colour-coded for length.

### Histological analyses

For histological analyses, the specimen was cut into two pieces approximately in the thorax region to ensure proper resin infiltration. The obtained pieces were afterwards dehydrated in an acidified dimethoxypropane solution, followed by several rinses in pure acetone before infiltration started in Agar Low Viscosity resin (Agar, Stansted, Essex, UK). After embedding and polymerization of the resin, ribbons of serial sections were conducted with a Diatome HistoJumbo diamond knife (Diatome, Switzerland). Sections were stained with toluidine blue, sealed and afterwards photographed and analysed with a Nikon NiU compound microscope equipped with a Nikon DsRi2 microscope camera (Nikon, Tokyo, Japan). Serial sections were converted into greyscale using FIJI^43^ and afterwards imported into the 3D visualization software Amira (Thermo Fisher). Images were aligned using the AlignSlices module of Amira. Afterwards, virtual sections of the CT stack of the parasitized and reconstructed specimen were placed using the histological sections as reference in order to compare the CT information with the histological details.

### Ethical approval

The government of the Netherlands authorized admission and rehabilitation procedure of different seal species at the Sealcentre Pieterburen (permission ID at time of sample-taking: FF/75/2012/015).

## Supplementary Information

Supplementary Information 1.

Supplementary Video 1.

## Data Availability

The datasets generated during and/or analyzed during the current study are available from the corresponding author on reasonable request.
