# Supplementary material for: Novel 3D in situ visualization of seal heartworm (Acanthocheilonema spirocauda) larvae in the seal louse (Echinophthirius horridus) by X-ray microCT
Source: Sci Rep. 2022 Aug 18;12:14078. doi: 10.1038/s41598-022-18418-y (PMC9388652; doi:10.1038/s41598-022-18418-y)
Supplement: Supplementary file 1 — Supplementary Information 1. [file 41598_2022_18418_MOESM1_ESM.pdf]

Supplementary material

Supplementary Video 1.mpg. Video of 3D reconstruction of *Echinophthirius horridus* showing various colored larval stages of *Acanthocheilonema spirocauda*. In situ visualization.
